# Editorial Announcements

**Published:** 1913-11

**Authors:** 


### Editorial Announcements

*To the Physicians of Auburn*: A sample copy of this issue is mailed to every physician in your city, aside from those included in the regular subscription list. We ask you to note that this Journal is especially devoted to the medical profession of Western New York, as indicated by the Associate Staff on the front cover. We aim, in every respect, to serve all parts of this region impartially. Please note the general contents of the Journal, and, especially, the headings of the various departments and the fact that a special editor has been appointed to represent your city. Your assistance in furnishing personal and general news items is requested.

At the risk of seeming to have interested motives, it may be admitted that we would like to secure more subscribers. There is no objection to sharing subscriptions.

